# Simulation Study on Residual Stress Distribution of Machined Surface Layer in Two-Step Cutting of Titanium Alloy

**DOI:** 10.3390/ma17174283

**Published:** 2024-08-29

**Authors:** Jingyi Wang, Bo Kong, Shulei Wei, Jian Zang, Anhai Li

**Affiliations:** 1 Key Laboratory of High Efficiency and Clean Mechanical Manufacture of MOE, School of Mechanical Engineering, Shandong University, Jinan 250061, China; wjy6660820@163.com (J.W.); kongbo.sripe@sinopec.com (B.K.); jianzang0824@163.com (J.Z.); 2 Shandong Engineering Research Center for Downhole Cementing and Completion Tools in Petroleum Engineering, Shelfoil Petroleum Equipment & Services Co., Ltd., Dezhou 253034, China; weishl.sripe@sinopec.com

**Keywords:** finite element analysis, titanium alloy, two-step cutting, high-speed cutting, residual stress

## Abstract

Ti-6Al-4V titanium alloy is known as one of the most difficult metallic materials to machine, and the machined surface residual stress distribution significantly affects properties such as static strength, fatigue strength, corrosion resistance, etc. This study utilized finite element software Abaqus 2020 to simulate the two-step cutting process of titanium alloy, incorporating stages of cooling, unloading, and de-constraining of the workpiece. The chip morphology and cutting force obtained from orthogonal cutting tests were used to validate the finite element model. Results from the orthogonal cutting simulations revealed that with increasing cutting speed and the tool rake angle, the residual stress undergoes a transition from compressive to tensile stress. To achieve greater residual compressive stress during machining, it is advisable to opt for a negative rake angle coupled with a lower cutting speed. Additionally, in two-step machining of titanium alloy, the initial cutting step exerts a profound influence on the subsequent cutting step, thereby shortening the evolution time of the Mises stress, equivalent plastic strain, and stiffness damage equivalent in the subsequent cutting step. These results contribute to optimizing titanium alloy machining processes by providing insights into controlling residual stress, ultimately enhancing product quality and performance of structural part of titanium alloy.

## 1. Introduction

Titanium alloys, recognized for their significant application potential, boast high specific strength, excellent heat resistance, and superb corrosion resistance. Since the mid-20th century, these alloys have been extensively used in various industries, including aerospace, shipbuilding, medical, metallurgy, and chemical sectors [1]. Despite their numerous advantageous properties, titanium alloys pose challenges in machining due to low thermal conductivity, high chemical activity, and a small deformation coefficient. These characteristics often result in chip serration, increased cutting force, and accelerated tool wear during machining, hindering the broader application of titanium alloys. Machining titanium alloys involves complex phenomena such as tribology, elastic-plastic mechanics, and fracture mechanics. The process is characterized by high temperatures, high strain, high strain rate, and the presence of thermal-force coupling, all occurring over short time periods [2]. Consequently, machining surfaces inevitably develop residual stress, which significantly impacts the mechanical properties of titanium alloys, including strength, hardness, and toughness. These stresses can also increase surface roughness and reduce the fatigue life of parts. Therefore, studying the magnitude of residual stress and the factors influencing it is crucial for improving the machining processes of titanium alloys.

Measuring and studying the influencing factors of residual stress using experimental methods is both time-consuming and labor-intensive, increasing research costs and complicating the acquisition of local stresses and strains during the machining process. Consequently, simulation models are predominantly used to study the variation of residual stresses. Wang et al. [3] established a three-dimensional numerical model to predict surface residual stress in multi-axis milling of Ti-6Al-4V titanium alloy and conducted experimental validation, finding average absolute errors of 11.6% for σ*_x_* and 15.2% for σ*_y_*. Wu et al. [4] created a three-dimensional finite element model using ABAQUS to analyze chip formation, stress distribution, cutting forces, and milling temperatures during the complex milling process of Ti-6Al-4V. Dong et al. [5] applied a bimodal Gaussian function to fit the residual stress distribution obtained from a finite element model for Inconel 718 alloy, investigating the impact of cutting parameters on residual stress distribution and identifying cutting speed as the most influential factor. Ren et al. [6] utilized a three-dimensional finite element model based on the Johnson-Cook ontological model of Ti-6Al-4V, combined with a modified Coulomb friction stress model, to analyze defects in laser additive manufacturing metal parts with large residual stresses. Li et al. [7] used the FEM to characterize the kinematics of three-dimensional elliptical vibratory cutting, demonstrating that this method facilitates the acquisition of compressive stress near the machined surface, thereby improving performance. 

Typically, residual tensile stress causes cracks on the machined surface, which in turn reduces the fatigue strength, while residual compressive stress can offset some of the tensile stress exerted by the working load, which in turn improves the fatigue life. The magnitude of residual stress is usually influenced by cutting parameters, tool geometry, workpiece shape, and material properties. Outeiro et al. [8] predicted residual stresses under different cutting conditions using a machine learning method based on mathematical regression analysis. Their results indicated that to increase compressive residual stress on the machined surface by 40%, the rake angle should be increased from −6° to 5°, and cutting speed should be reduced by 67% (from 60 m/min to 20 m/min). Yao et al. [9] found that surface residual compressive stress increases with the rake angle of the cutting tool during high-speed milling of titanium alloy TC11. Dehmani [10] et al. developed a numerical model of orthogonal cutting and investigated the effect of tool edge radius and heat generated by friction on the flank face on residual stress. It was shown that the impact of heat on residual stress could not be overlooked. Sun et al. [11] experimentally demonstrated that as cutting speed increases, the compressive residual stress in both the cutting and feed directions intensifies, with the residual compressive stress in the feed direction being approximately 30% greater than that in the cutting direction. Matuszak et al. [12] observed that the maximum residual compressive stress and its thickness peaked at a cutting speed of 190 m/min.

In practical machining, multistep cutting has garnered significant attention due to its impact on surface residual stress. Zhang et al. [13] demonstrated that cutting force and residual compressive stress decrease with increasing roughing cutting speed in two-step milling tests on Ti-6Al-4V titanium alloy. Song et al. [14] simulated and analyzed various cutting processes using a finite element simulation model and found that multistage cutting can increase compressive residual stress and alter the location of minimum residual stress along the depth direction. They also discovered that pre-stressing multistage cutting maximizes the compressive residual stress value. Liu and Guo [15] used finite element software to simulate multistage cutting and employed thermo-viscoelastic finite element simulations to study the effects of cutting force unloading and clamping forces on residual stress distribution. Aassif et al. [16] studied the influence of temperature and strain accumulation on the residual stress distribution of the subsequent machining process.

Currently, both domestic and international scholars primarily use a combination of experimental and simulation methods to study residual stress in titanium alloy machining. However, most simulation models for a single step do not account for the significant heat generated in the first high-speed cutting step or the impact of residual stress on the second cutting step, while the actual machining is usually a multi-step process. In this paper, we establish a two-dimensional cutting simulation model using the finite element method and set up key technologies such as cooling stage and workpiece material unloading and de-constraint, which solves the effects of residual heat and stress of the previous process on the newly machined surface and is more in line with the actual machining. We explore the effects of different tool angles and cutting speeds on residual stress and the reasons for changes in residual stress under various conditions. Additionally, we innovatively analyze the effects of the first cutting step on the evolution of stress, strain, and stiffness damage over time in the second cutting step, demonstrating the complex interactions between the steps in multistep machining. The study's findings can be used to evaluate the effects of tool rake angle, cutting speed, and different working steps on residual stress in the cutting process, providing practical insights for controlling residual stress and enhancing process optimization in actual machining.

## 2. Materials and Methods

**Figure 1:**
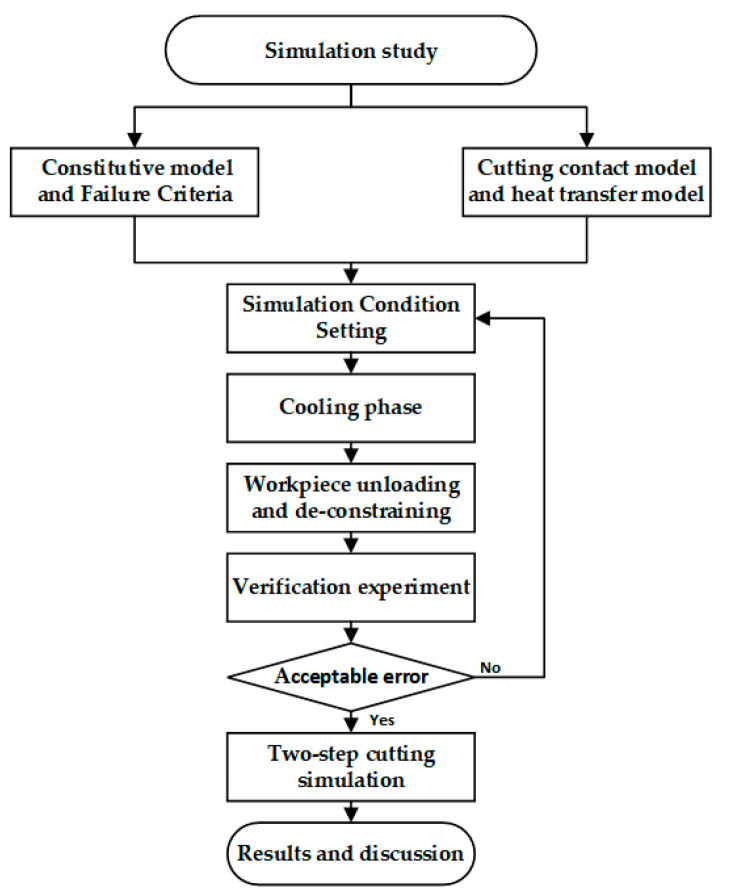
The flowchart of the simulation study.

This section will describe the materials and equipment used for simulation and experiments, and Figure 1 shows the flowchart of the simulation design and validation experiments.

### 2.1. Simulation Condition Setting

**Figure 2:**
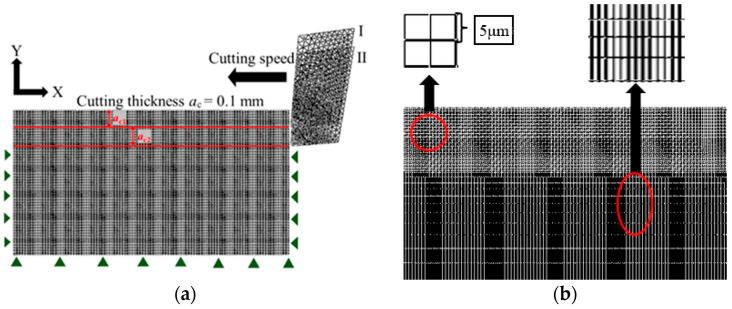
Multi-step cutting finite element simulation model. (**a**) Multi-step cutting model; (**b**) Workpiece meshing model.

In the simulation test, a Ti-6Al-4V multistep cutting model was established using simulation software Abaqus 2020. The finite element model was configured as a two-dimensional orthogonal cutting model [17], as illustrated in Figure 2a. A simplified integral four-node bilinear thermodynamic coupling unit [18] was used for the workpiece. During the simulation, the tool was modeled as a rigid body due to its minimal deformation. The degrees of freedom at the bottom edge of the workpiece in both horizontal and vertical directions, as well as the horizontal direction of the side edges, were constrained to prevent displacement caused by the tool's motion (the green arrowheads in Figure 2a). The boundary temperature was set to room temperature, and a reference point was selected on the right side of the tool to which the cutting speed was applied. The tool angle and cutting parameters in the model are detailed in Table 1. Simulation trials No.1–No.4 are used to compare the results with those of the validation experiments, No.5–No.11 are used to study the effect of tool rake angle on the machined residual stress, No. 9 and No.12–14 are used to study the effect of cutting speed on the residual stress and the machined surfaces, and No.15–No.18 are used to study the effect of two-step cutting on the evolution of the machined surface layer state. To improve the efficiency of the cutting simulation, Figure 2b depicts the grid cell division of the workpiece. The mesh size of the layer to be cut was set to 5 × 5 μm, while the material matrix part was configured with a dimensionally gradual mesh.

### 2.2. Constitutive Model and Failure Criteria

The ontological relationships of materials are fundamental for describing their dynamic mechanical behavior. During machining, the deformation of workpiece materials typically involves high strains, high strain rates, and elevated temperatures. The Johnson–Cook material model, which integrates the effects of strain, strain rate, and temperature on flow stress, accommodates high strain rates ranging from 10^2^ to 10^6^ [19]. Consequently, it is frequently used in finite element cutting simulation models. The expression of the Johnson-Cook material model is as follows [20].

(1) $$\sigma =\left(A+B\varepsilon^{n}\right)\left(1+C\ln\frac{\dot{\bar{\varepsilon }}}{{\dot{\bar{\varepsilon }}}_{0}}\right)\left[1-{\left(\frac{T-T_{r}}{T_{m}-T_{r}}\right)}^{m}\right]$$

where σ is the flow stress of the workpiece material; ε is the equivalent plastic strain of the workpiece material; ε is the equivalent plastic strain of the workpiece material; $\frac{\dot{\bar{\varepsilon }}}{{\dot{\bar{\varepsilon }}}_{0}}$ is the dimensionless plastic strain rate; ${\dot{\bar{\varepsilon }}}_{0}$ is the reference strain rate; *T*_r_ is the room temperature (20 °C); *T*_m_ is the melting temperature of the material.

Table 2 shows the specific values of the parameters of the Johnson-Cook material model for titanium alloys. Table 3 and Table 4 show the performance parameters and main chemical composition of titanium alloys.

In this paper, the Johnson-Cook failure model [23] is used to define the damage parameters based on the equivalent plastic strain at the integration point of the unit, expressed as Equation (2).

(2) $$w=\sum \frac{\Delta \bar{\varepsilon }}{{\bar{\varepsilon }}_{f}}$$

where w is workpiece material damage parameter; $\Delta \bar{\varepsilon }$ is increment of equivalent strain of workpiece material; ${\bar{\varepsilon }}_{f}$ is equivalent strain of workpiece material. Equivalent strain ${\bar{\varepsilon }}_{f}$ is expressed as Equation (3).

(3) $${\bar{\varepsilon }}_{f}=\left[D_{1}+D_{2}\exp\left(D_{3}\frac{P}{\bar{\sigma }}\right)\right]\left[1+D_{4}\ln\frac{\dot{\bar{\varepsilon }}}{{\dot{\bar{\varepsilon }}}_{0}}\right]\left[1+D_{5}\frac{T-T_{r}}{T_{m}-T_{r}}\right]$$

where *P* is the average value of the three principal stress. $\bar{\sigma }$ is the equivalent stress. *D*_1_~*D*_5_ is material failure parameters. For titanium alloy materials, the specific values of these failure parameters are provided in Table 5. During the simulation, the finite element software accumulates the failure parameters at the end of each analysis step. If the damage parameter *w* exceeds 1, the mesh element is considered to have failed and is removed from the overall mesh.

The stress-strain process of deformation damage in materials during cutting can be divided into three stages [25]. The first stage is elastic deformation, where stress gradually increases. Once the stress exceeds the yield stress $\sigma_{0}$, the material enters the stable plastic deformation stage, during which strain hardening is more significant than thermal softening. When the damage parameter *w* reaches 1, damage begins to appear, and the damage variable *D* starts to increase from 0, marking the onset of the damage evolution stage. At this point, thermal softening becomes dominant, strain increases, and stress decreases until the material completely fails (*D* = 1) and the stress drops to 0.

### 2.3. Cutting Contact Model and Heat Transfer Model

When machining titanium alloys, friction in the tool-workpiece contact zone significantly affects tool life, cutting heat, and the quality of the machined surface. In the cutting simulation model, it is crucial to accurately represent material deformation and the friction between the tool and the chip. Friction in the cutting process primarily occurs between the tool’s rake face and the chip, as well as between the flank face and the machined surface. The modified Coulomb friction model [26] is used to define the friction properties. The friction region between the chip and the tool's rake face is divided into two segments: the bonding zone, where the material experiences shear stress $\tau_{f}$ approximately equal to its shear yield strength $\tau_{\gamma }$, and the sliding zone, where the friction stress is proportional to the normal stress $\sigma_{n}$, with the proportionality coefficient being the friction factor. In the model, the friction factor μ is set to 0.3 [27].

(4) $$\tau_{f}=\left\{\begin{array}{l} \tau_{\gamma },\tau_{\gamma }\leq \mu \sigma_{n}\\ \mu \sigma_{n},\tau_{\gamma }>\mu \sigma_{n} \end{array}\right.$$

During the cutting process, a large amount of cutting heat is generated due to the friction in the tool-chip and tool-workpiece contact zones as well as the deformation of the material, and it is mainly concentrated in the shear zone and the tool-chip contact zone. Its heat transfer control equation is [28].

(5) $$\lambda \left(\frac{\partial^{2}T}{\partial^{2}x}+\frac{\partial^{2}T}{\partial^{2}y}\right)+\dot{Q}=\rho C_{p}\left(u_{x}\frac{\partial T}{\partial x}+u_{y}\frac{\partial T}{\partial y}\right)$$

*T* is the temperature as a function of *x* and *y* in a two-dimensional plane. *λ* represents thermal conductivity, and $\dot{Q}$ denotes heat flow per unit volume. The transfer velocity of the moving heat source is $u_{x}\;$in the *x* direction and $u_{y}$ in the *y* direction. 

Calculation of heat due to plastic deformation of materials

(6) $${\dot{Q}}_{p}=\eta_{p}\bar{\sigma }\cdot \bar{\varepsilon }/J$$

where ${\dot{Q}}_{p}$ is volumetric heat flow rate from plastic deformation; $\eta_{p}$ is plastic deformation work conversion coefficient, set to 0.9 [29]; *J* is thermal work equivalence coefficient; $\bar{\sigma }\;$is equivalent force of the material in the cutting process; $\bar{\varepsilon }$ is equivalent strain of the material in the cutting process.

Calculation of heat generated due to tool-chip friction

(7) $${\dot{Q}}_{f}=\eta_{f}\tau_{f}v_{chip}/J$$

where ${\dot{Q}}_{f}$ is volumetric heat flow rate from friction; $v_{chip}$ is tool-chip relative rate; $\eta_{f}$ is friction work conversion coefficient.

The frictional work conversion factor in the model is set to 0.5 [30], which indicates that the heat carried away by the tool and chip each accounts for 50% of the heat generated by friction.

### 2.4. Cooling Phase

**Figure 3:**
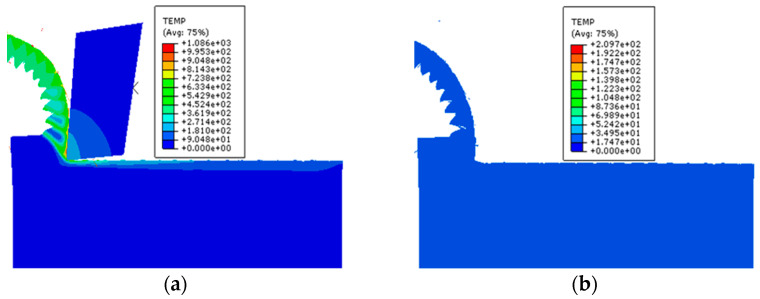
Temperature distribution nephogram (**a**) workpiece before cooling; (**b**) workpiece after cooling.

**Figure 4:**
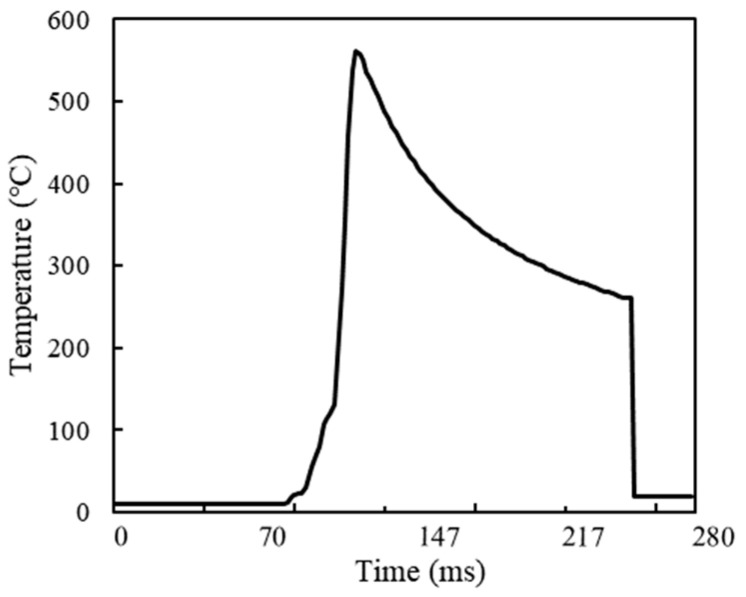
Temperature change of machined surface material.

Due to the tool-workpiece interaction, the surface temperature of the workpiece increases after each machining pass, causing some material softening. This softening can affect cutting results and the extraction of cutting process data if the next step in the cutting simulation is carried out immediately. Figure 3 illustrates the temperature distribution nephogram of the material after machining. Figure 3a shows that the substantial heat generated during machining raises the surface temperature of the workpiece, making it unsuitable for immediate subsequent cutting. Conversely, Figure 3b demonstrates a significant decrease in workpiece temperature after the cooling stage, which is crucial for ensuring the accuracy of subsequent cutting and machining results. Figure 4 depicts the temperature change curve of the machined surface post-machining. It is evident that the surface temperature reaches its peak during machining; upon completion, the boundary condition of room temperature is set in the model. The surface temperature gradually decreases to room temperature, with a time interval of 1.75 ms between each data point. Proceeding with subsequent machining at this stage will significantly reduce the impact of the first step on the second.

### 2.5. Workpiece Unloading and De-Constraining

**Figure 5:**
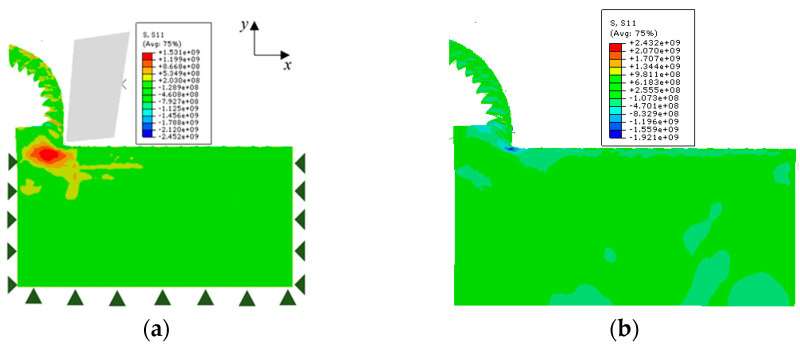
Stress distribution of workpiece (**a**) before de-constraining; (**b**) after de-constraining.

As can be seen in Figure 5a, there is a large amount of stress on the surface after machining is complete, and eliminating stress is critical for subsequent machining. In the simulation model, the unloading process is primarily divided into tool action unloading and workpiece constraint removal. After each machining process, the tool retracts away from the workpiece. From the start to the end of the tool-workpiece contact, the boundary conditions remain unchanged. The workpiece is de-constrained to eliminate the influence of boundary conditions on its free deformation. Figure 5b displays the stress distribution in the *S*_11_ direction (x direction) before and after unloading the tool action and removing workpiece constraints following the completion of cutting. The stress is released after de-constraining the workpiece, resulting in a more uniform stress distribution.

### 2.6. Verification of the Simulation Model

#### 2.6.1. Setting of Verification Experiment

**Figure 6:**
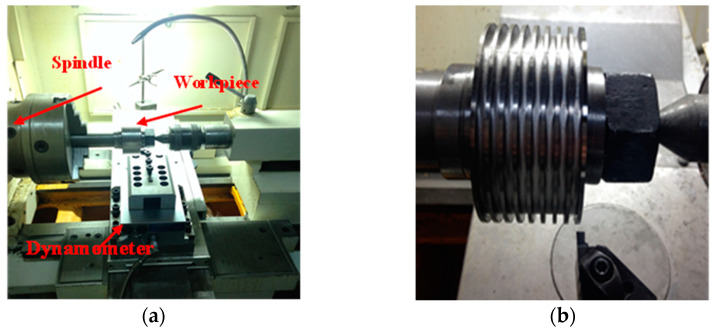
Experimental equipment in orthogonal cutting of Ti-6Al-4V titanium alloy (**a**) experimental equipment; (**b**) workpiece.

The orthogonal cutting test of titanium alloy was conducted on a CKD6143H CNC lathe (made by Shandong University, Jinan, China), using a NG3156R KC5025 TiAlN coated carbide tool (made by Kennametal Inc., Latrobe, PA, USA). The tool featured a rake angle of 0°, a flank angle of 7°, and a cutting-edge width of 3.96 mm. The workpiece material was Ti-6Al-4V titanium alloy, with its main chemical composition listed in Table 4. The material diameter was 100 mm. The titanium alloy bar was machined into a ring-shaped groove with a width of 2 mm and a spacing of 3 mm by wire-cutting. The experimental equipment and measurement system in cutting tests are shown in Figure 6, and the cutting parameters are detailed in Table 6. Cutting forces during orthogonal turning were measured using a Kistler 9257B dynamometer (made by Kistler Group, Winterthur, Switzerland). Each set of tests was carried out three times, all with new tools to eliminate the errors introduced by tool wear on the cutting results.

Upon completion of each test, the chips were collected and labeled according to the cutting parameters. The chips were set, ground, polished, and etched using a corrosive solution composed of 3 ml hydrofluoric acid, 5 ml nitric acid, and 100 ml water, with a typical corrosion time of 30 to 60 s. A scanning electron microscope was used to observe the chip morphology, with the degree of serration in the serrated chips denoted as *G*_s_ [16]

(8) $$G_{s}=\frac{H-C}{H},$$

where *H* is the height of top of tooth and *C* is the height of tooth valley.

#### 2.6.2. Comparison of Cutting Forces

**Figure 7:**
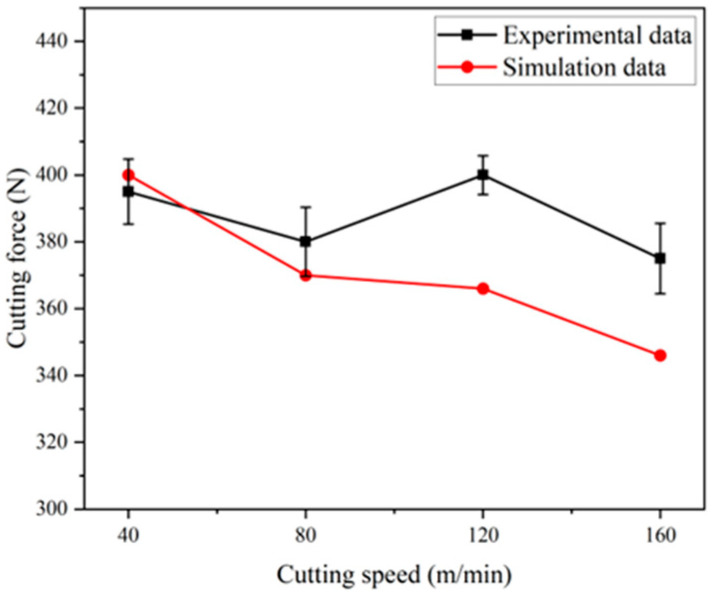
Comparison of cutting forces between test and simulation.

Using the established two-dimensional simulation model, cutting simulations were performed with a feed rate of 0.1 mm/rev, a depth of cut of 0.1 mm, and cutting speeds of 40 m/min, 80 m/min, 120 m/min, and 160 m/min. Figure 7 shows the cutting force obtained from the simulation and those measured during the tests. It can be observed that due to the wear of the turning test tool and the measurement error of the instrument, the cutting forces measured at cutting speeds of 80 m/min, 120 m/min, and 160 m/min are slightly higher than those in the simulation. At a cutting speed of 40 m/min, the measured cutting force is lower than the simulated cutting force. The relative errors are within 15%, demonstrating that the established simulation model has high prediction accuracy.

#### 2.6.3. Comparison of Chip Morphology

**Figure 8:**
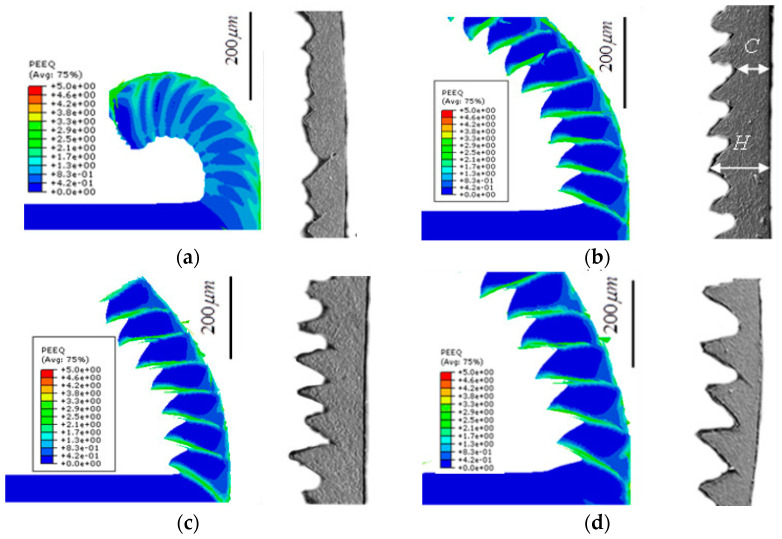
Comparison of chip morphology obtained from simulation and experiment (**a**) *v* = 40 m/min; (**b**) *v* = 80 m/min; (**c**) *v* = 120 m/min; (**d**) *v* = 160 m/min.

**Figure 9:**
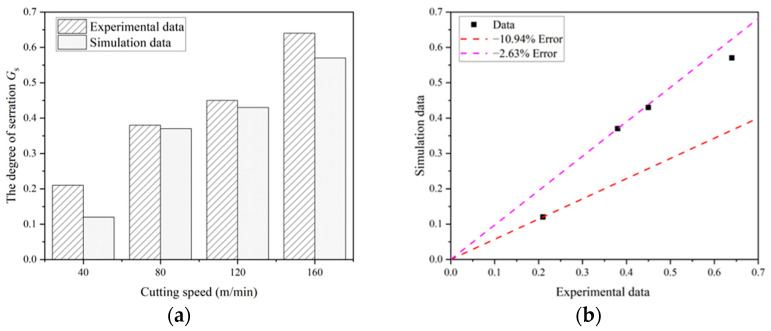
Degree of chip serration *G_s_* obtained from simulation and experiment (**a**) trend of chip serration degree with cutting speed; (**b**) simulated and experimental chip serration degree error.

Figure 8 compares the chip morphology obtained from the cutting simulation and the test, while Figure 9 illustrates the trend of chip serration degree from both the simulation and the test at various cutting speeds, along with the relative error between the two methods. It is evident that the chip morphology in both the simulation and the test shows consistent trends under different cutting parameters. At a cutting thickness of 0.1 mm and a cutting speed of 40 m/min, both the simulation and the test produce band-shaped chips. As the cutting speed increases to 80 m/min, 120 m/min, and 160 m/min, the chip morphology transitions from band-shaped to sawtooth-shaped, with the degree of serration increasing with the cutting speed. Errors in the degree of serration ranged from −2.63% to −10.94%. The similarity in chip shape between the simulation and the test directly reflects the accuracy and effectiveness of the simulation model.

## 3. Results and Discussion

### 3.1. Selection of Residual Stress Direction

**Figure 10:**
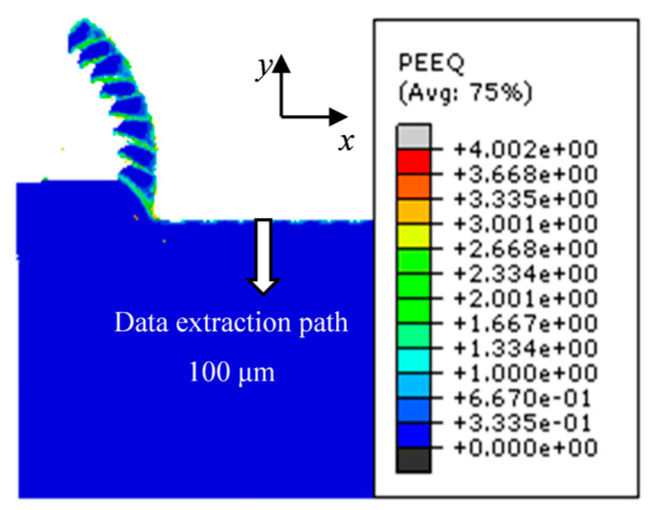
Residual stress data extraction path.

**Figure 11:**
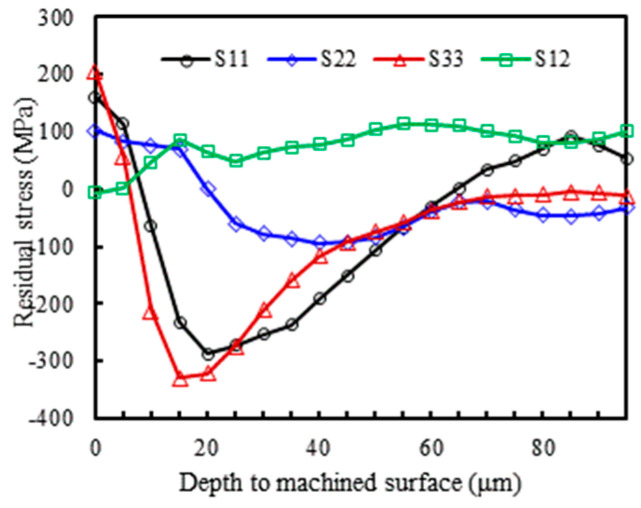
Variation of residual stress in all directions along the depth to the machined surface.

To extract residual stress data from the cutting model in all directions along the depth, the data extraction path is shown in Figure 10. The depth for residual stress extraction is 100 μm. At a cutting speed *v* of 400 m/min, a tool rake angle *γ* of 5°, and a cutting thickness *a*_c_ of 0.1 mm, the variation of residual stress in each direction with the depth of the workpiece surface layer is illustrated in Figure 11. In this figure, *S*_11_, *S*_22_, and *S*_33_ represent the stress along the *X*, *Y*, and *Z* axes, respectively, while *S*_12_ represents the shear stress along the *Y* direction on the *XY* plane. Positive values indicate tensile stress, and negative values indicate compressive stress. As shown in Figure 11, with increasing depth from the machined surface, the fluctuation range of *S*_11_ and *S*_33_ is larger and more evidently discernible. Therefore, the residual stress in the *S*_11_ and *S*_33_ directions are selected as the focus to study the influence of tool rake angle and cutting speed on their behavior.

### 3.2. Effect of Tool Rake Angle on Machining Residual Stress

**Figure 12:**
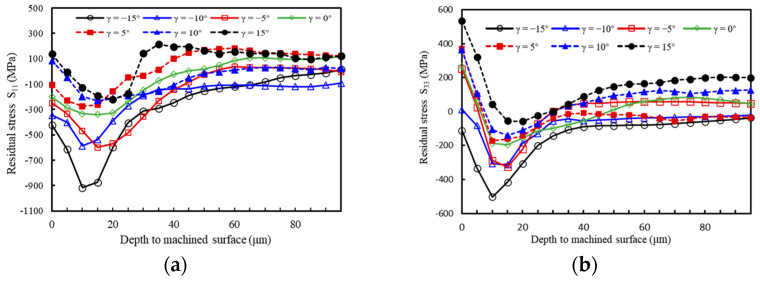
Variation of machining residual stress along the depth to the machined surface at different tool rake angles (**a**) *S*_11_ direction; (**b**) *S*_33_ direction.

In the machining of titanium alloy, the distribution of residual stress in the depth direction from the machined surface is shown in Figure 12 when the tool rake angle is varied in the simulation model at a cutting speed of 200 m/min. From Figure 12a, it is evident that the distribution pattern of residual stress in the depth direction changes significantly with increasing tool rake angle. When the rake angles are −15°, −10°, −5°, 0°, and 5°, the surface material exhibits residual compressive stress in the *S*_11_ direction, and decreases with the increase in tool rake angle; when the tool rake angle is 10° and 15°, it presents surface residual tensile stress and increases gradually. At a tool rake angle of 15°, the tensile stress reaches 137 MPa. In Figure 12b, for the *S*_33_ direction, the machined surface is under compressive stress when the tool rake angle is −15°. As the tool rake angle increases, the machined surface residual stress shifts to tensile stress and gradually increases; when the tool rake angle is 15°, the tensile stress is the largest, at 535 MPa. The total peak residual compressive stress decreases with the increase in the rake angle. At the same time, it can be seen from Figure 12 that in the case of different tool rake angles, the distribution pattern of residual stress with the depth of the machined surface shows consistency, and the compressive stress increases first and then decreases.

The reason for this phenomenon is that during the cutting process, when the tool rake angle is small, the cutting edge radius is larger, and the extrusion and friction of the tool on the machined surface is greater. The machined surface is primarily influenced by mechanical load, resulting in residual compressive stress [31]. As the tool rake angle increases, the influence of thermal load on the machined surface becomes stronger than that of the mechanical load, leading to residual tensile stress. Additionally, as shown in Figure 12, the distribution of residual stress with the depth of the machined surface exhibits a consistent pattern across different tool rake angles. In practice, a negative rake angle can be selected to obtain residual compressive stress and thus reduce chipping.

### 3.3. Effect of Cutting Speed on Machining Residual Stress and Machined Surface

With other cutting parameters held constant, the residual stress in the depth direction were investigated using the finite element model at different cutting speeds. In this study, the tool rake angle was 5°, the cutting thickness was 0.1 mm, and the cutting speeds were 100 m/min, 200 m/min, 300 m/min, and 400 m/min.

**Figure 13:**
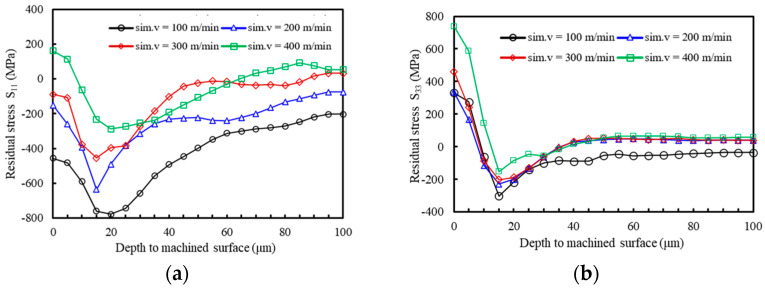
Variation of machining residual stress along the depth to the machined surface at different cutting speed (**a**) *S*_11_ direction; (**b**) *S*_33_ direction.

The residual stress distribution on the machined surface is shown in Figure 13, revealing that the surface material presents a compressive stress state in the direction of S_11_, when the cutting speed is small. Within a certain range of cutting speeds, the surface tensile stress increases as the cutting speed increases, and the maximum tensile stress is 161 MPa when the cutting speed is 400 m/min. While in the direction of S_33_, the surface residual stress presents a tensile stress state, and it also increases with the increase in speed. The total peak residual compressive stress decreases with the increase in cutting speed. This trend is consistent with the results in the study by Yang et al. [32] and proves the accuracy of the model. This trend is primarily due to the increased thermal load on the machined surface at higher speeds, which gradually outweighs the mechanical load effect [33], resulting in residual tensile stress.

As the surface depth increases, the tensile stress decreases within the 0~15 μm range, and the slope of the corresponding curve increases sequentially with cutting speed. The primary reason for this behavior is that higher cutting speeds lead to increased strain rates in the processed surface material, thus increasing the compressive stress. However, as the depth of the surface layer increases, heat conduction diminishes, reducing the thermal load effect and enhancing the mechanical load effect. Plastic deformation of the material becomes dominant, and the stress state transitions to compressive stress [34]. The minimum values of residual tensile stress at different cutting speeds occur at nearly the same depth, indicating that mechanical and thermal loads have a minimal impact on the depth distribution of residual stress during the cutting process. Beyond a certain depth, the residual stress values tend to zero, primarily because the effects of mechanical and thermal loads diminish with increasing depth.

**Figure 14:**
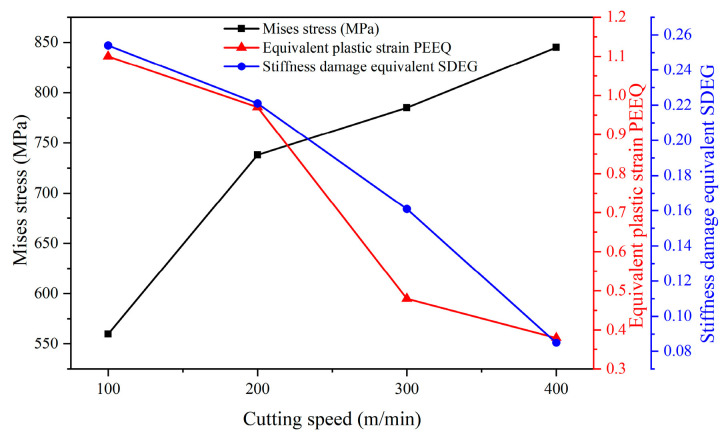
Variation of stress, strain, and stiffness damage equivalent with cutting speed on machined surfaces in the first cutting step.

Figure 14 shows the changes in stress, strain, and stiffness damage equivalent with varying cutting speeds during the first cutting step, revealing the effect of cutting speed on surface machining performance. It can be seen that as the cutting speed in the first cutting step increases, the Mises stress gradually increases, while the equivalent plastic strain and stiffness damage equivalent gradually decrease.

### 3.4. Effect of Two-Step Cutting on the Evolution of the Machined Surface Layer State

**Figure 15:**
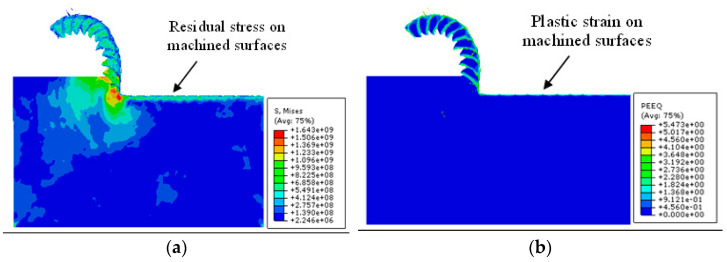
Multi-step cutting simulation results (**a**) Mises stress distribution; (**b**) equivalent plastic strain distribution.

Due to the interaction between the tool and the workpiece, the stress, strain, and stiffness damage equivalent of the machined surface change after the first cutting step of machining, subsequently affecting the surface quality of the second step. Figure 15 shows the nephogram of the stress and strain distribution on the workpiece surface after the first machining step. It can be observed that there are residual stress and plastic strains on the machined surface of the workpiece material.

**Figure 16:**
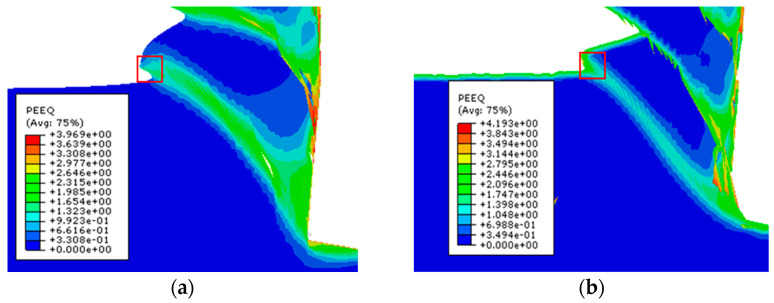
Workpiece surface reference unit (**a**) surface reference unit for the first cutting step; (**b**) surface reference unit for the second cutting step.

The stress-strain curves indicate that the stress, strain, and damage evolution of the material can describe the deformation process in greater detail. To investigate the effects of stress, strain, and stiffness damage equivalent on the machined surface of the first cutting step on the machined surface of the second cutting step, O points in the adiabatic shear band were marked before both the first and second cutting steps of machining, as shown in Figure 16. Data extraction for Mises stress, equivalent plastic strain, and stiffness damage equivalent was performed during the formation of the adiabatic shear zone, with a time interval of 1.75 ms between each data point.

**Figure 17:**
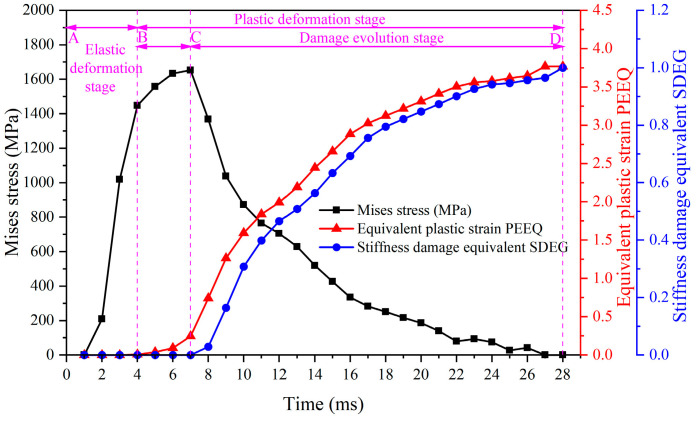
Evolution of stress, strain, and stiffness damage equivalent with time for the reference unit of the first cutting step.

Figure 17 shows the evolution of plastic strain, stress, and stiffness damage equivalent of the reference unit over time during the first cutting step. The cutting speed for both the first and second cutting steps is 200 m/min, and the cutting thickness is 0.1 mm. It can be seen that in the AB stage, the material is in the elastic deformation phase. Under the action of the cutting tool, the internal stress of the reference unit gradually rises, while the plastic strain and stiffness damage equivalent remain at 0. In the BC stage, as the stress within the unit increases, the material enters the plastic deformation phase. The stress gradually increases to the yield stress, and the reference unit plastic strain begins to increase, reaching 0.245 at point C. When the unit stress reaches its peak at the yield stress, it begins to decrease in the CD section, while the reference unit strain continues to increase. When the unit stress decreases to zero, the strain reaches a maximum value of 3.768. At point C, when the stress reaches its maximum value, the stiffness damage equivalent begins to rise. As the stiffness damage equivalent rapidly increases to 1, the reference unit fails, and the stress becomes 0, calling the CD stage as the damage evolution phase.

**Figure 18:**
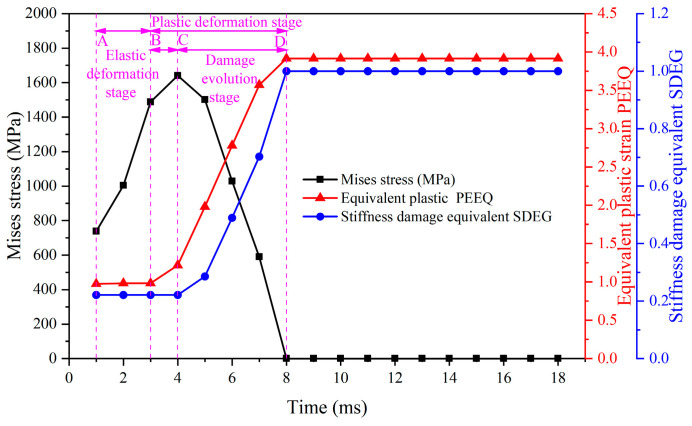
Evolution of stress, strain, and stiffness damage equivalent with time for the reference unit of the second cutting step.

Figure 18 shows the evolution of stress, strain, and stiffness damage equivalent over time in the reference cell of the chip's free surface obtained in the second cutting step. The evolution patterns in the second cutting step, including stress, plastic strain, and stiffness damage equivalent, are similar to those in the first cutting step, encompassing elastic deformation, plastic deformation, and damage evolution stages. The figure indicates that, due to the influence of the first cutting step, the initial values of stress, strain, and stiffness damage equivalent in the reference unit of the second cutting step differ from those in the first cutting step. The initial stress is 738 MPa, the strain is 0.97, and the stiffness damage is 0.221. At the end of the damage evolution stage, the strain reaches 3.914. This variation is primarily due to the friction and extrusion effects of the tool flank face and cutting edge on the machined surface during the first cutting step, which induce residual stress and generate substantial cutting heat. The heat-force coupling effect leads to stiffness damage on the machined surface. From the stiffness damage equivalent change curve over time, it is evident that the stiffness damage rate in the reference unit during the second cutting step is significantly higher than in the first cutting step. The stiffness damage equivalent reaches 1 in a much shorter time, resulting in the failure of the reference unit.

By comparing Figure 17 with Figure 18, it can be seen that under the same machining parameters in both the first and second cutting steps, the evolution time to failure of the workpiece surface unit in the second cutting step is substantially shorter than in the first cutting step. The time taken for the stress in the reference unit of the first cutting step to reach its maximum point is longer than the evolution time in the second cutting step. Additionally, during the damage evolution stage, the rate of stress decrease in the reference unit of the second cutting step is significantly greater than that in the first cutting step.

### 3.5. Limitations and Outlook

Despite the progress made in this study, there are some limitations. In the cutting simulation, only the effects of different machining speeds and rake angles on residual stress were discussed, while no further analysis was performed on the effects of other tool geometry parameters and cutting parameters such as cutting thickness. And there is no research on systematic optimization parameters based on the simulation results. To address these limitations, future research should broaden the experimental scope to encompass the influence of multiple factors and devise optimization algorithms tailored for multi-step titanium alloy cutting parameters. In addition, for the multi-step simulation model, we should attempt to directly simulate the second step of machining by changing the properties of stress, strain and stiffness damage equivalent to the material to be cut, so as to improve the simulation efficiency and reduce the modeling workload.

## 4. Conclusions

This paper utilizes FEM to simulate the effect of cutting speed and tool rake angle on the residual stress on the machined surface. Additionally, the simulation analyzes the impact of the first cutting step on the second cutting step.

(1) The two-dimensional orthogonal cutting model of titanium alloy was carried out using Abaqus. The material cooling phase, unloading, and de-constraints were included to enhance the accuracy of multi-step cutting simulation. The correctness of the simulation model was verified by an orthogonal cutting test on the titanium alloy, and the relative error of cutting force was within 15%. The errors of the degree of serration ranged from −2.63 to −10.94%.
(2) The effect of different cutting parameters on residual stress was analyzed using simulation models. The results show that the residual compressive stress decreases and the residual tensile stress increases gradually with the increase in tool rake angle. When the tool rake angle is 15°, the tensile stress grows to 137 Mpa. The residual stress with the increase in cutting speed shows a similar trend with the rake angle. With the increase in surface depth, the residual compressive stress first increases and then decreases and gradually disappears beyond a certain depth.
(3) By extracting data from the reference unit of the simulation model, the change in Mises stress, equivalent plastic strain, and stiffness damage equivalent was analyzed during two cutting steps. The initial values of Mises stress, PEEQ, and SDEG for the first cutting step are 0, while for the second cutting step, the initial Mises stress is 738 MPa, the PEEQ is 0.97, and the SDEG is 0.221. Under the same conditions, the first cutting step affects the initial values of indicators of the second cutting step, as well as the evolution time of them. As the cutting speed of the first cutting step increases, the Mises stress gradually increases, while the PEEQ and SDEG of the machined surface unit gradually decrease.

## Figures and Tables

**Table 1:** Simulation parameters for multi-step cutting of titanium alloys.

Sim. No.	Tool Rake Angle *γ* (°)	Cutting Speed of the First Step *v*_1_ (m/min)	Cutting Speed of the Second Step *v*_2_ (m/min)	Cutting Thickness *a_c_* (mm)
1	0	40	/	0.1
2	0	80		
3	0	120		
4	0	160		
5	−15	200		
6	−10	200		
7	−5	200		
8	0	200		
9	5	200		
10	10	200		
11	15	200		
12	5	100		
13	5	300		
14	5	400		
15	5	100	200	
16	5	200	200	
17	5	300	200	
18	5	400	200	

**Table 2:** Parameters of the Johnson-Cook constitutive model for Ti-6Al-4V titanium alloy [18].

*A* (MPa)	*B* (MPa)	*n*	*C*	*m*
782	498	0.28	0.028	1

**Table 3:** Physical and chemical properties of Ti-6Al-4V titanium alloy [21].

Density *ρ* (kg/m^3^)	Elastic Modulus *E* (GPa)	Poisson Ratio *μ*	Thermal Conductivity *λ* (W/m·K)	Specific Heat *C*_p_ (J/kg·K)
4430	109 (50 °C) 91 (250 °C) 75 (750 °C)	0.34	6.8 (20 °C) 7.4 (100 °C) 9.8 (300 °C) 11.8 (500 °C)	611 (20 °C) 624 (100 °C) 674 (300 °C) 703 (500 °C)

**Table 4:** Chemical composition of Ti-6Al-4V titanium alloy [22].

Elements	Ti	Al	V	Fe	Si	C	N	H	O
wt. %	Base	5.6	3.86	0.18	<0.01	0.02	0.023	<0.01	0.17

**Table 5:** Johnson-Cook Failure Parameters for Ti-6Al-4V Titanium Alloy [24].

*D*_1_	*D*_2_	*D*_3_	*D*_4_	*D*_5_
−0.09	0.25	−0.5	0.014	3.87

**Table 6:** Orthogonal cutting parameters of Ti-6Al-4V titanium alloy.

Cutting Parameters	Value
Cutting width (mm)	2
Cutting speed *v* (m/min)	40, 80, 120, 160
Feed *f* (mm/r)	0.05, 0.10, 0.15, 0.20

## Data Availability

The dataset used in this paper is available from the corresponding author upon request.

## Nomenclature

The following parameters and abbreviations are used in this manuscript.

γ	Tool rake angle
*v*_1_	Cutting speed of the first step
*v*_2_	Cutting speed of the second step
*a_c_*	Cutting thickness
*σ*	Flow stress of the workpiece material
ε	Equivalent plastic strain of the workpiece material
$\frac{\dot{\bar{\varepsilon }}}{{\dot{\bar{\varepsilon }}}_{0}}$	Plastic strain rate
${\dot{\bar{\varepsilon }}}_{0}$	Reference strain rate
*T_r_*	Room temperature (20 °C)
*T_m_*	Melting temperature of the material
*A*	Yield strength
*B*	Hardening modulus
*n*	Strain hardening exponent
*C*	Strain rate sensitivity coefficient
*m*	Thermal softening coefficient
*ρ*	Density
*E*	Elastic Modulus
*μ*	Poisson Ratio
*λ*	Thermal conductivity
*C*_p_	Specific Heat
w	Workpiece material damage parameter
$\Delta \bar{\varepsilon }$	Increment of equivalent strain of workpiece material
${\bar{\varepsilon }}_{f}$	Equivalent strain of workpiece material
*P*	Average value of the three principal stress
$\bar{\sigma }$	Equivalent stress
*D*_1_~*D*_5_	Material failure parameters
$\sigma_{0}$	Yield stress
$\tau_{f}$	Material experiences shear stress
$\tau_{\gamma }$	Material shear yield strength
$\sigma_{n}$	Normal stress
*μ*	Friction factor
*T*	Temperature
$\dot{Q}$	Heat flow per unit volume
$u_{x}$	Transfer velocity of the moving heat source in the *x* direction
$u_{y}$	Transfer velocity of the moving heat sourcein the y direction
${\dot{Q}}_{p}$	Volumetric heat flow rate from plastic deformation
$\eta_{p}$	Plastic deformation work conversion coefficient
*J*	Thermal work equivalence coefficient
$\bar{\varepsilon }$	Equivalent strain of the material in the cutting process
${\dot{Q}}_{f}$	Volumetric heat flow rate from friction
$v_{chip}$	Tool-chip relative rate
$\eta_{f}$	Friction work conversion coefficient
*f*	Feed
*G_s_*	The degree of serration
*H*	Height of top of tooth
*C*	Height of tooth valley
*S*_11_	Stress along the X direction
*S*_22_	Stress along the Y direction
*S*_33_	Stress along the Z direction
*S*_12_	Shear stress along the Y direction on the XY plane
*FEM*	Finite element method
*PEEQ*	Equivalent plastic strain
*SDEG*	Stiffness damage equivalent
